# Vagus nerve stimulation ameliorates L-NAME-induced preeclampsia-like symptoms in rats through inhibition of the inflammatory response

**DOI:** 10.1186/s12884-021-03650-7

**Published:** 2021-03-04

**Authors:** Linmei Zheng, Rong Tang, Lei Shi, Mei Zhong, Zhongyi Zhou

**Affiliations:** 1 Department of Obstetrics and Gynecology, Nanfang Hospital, Southern Medical University, Guangzhou, Guangdong China; 2 Department of department of General Surgery, Hainan General Hospital, Hainan Affiliated Hospital of Hainan Medical University, Haikou, Hainan China; 3 Department of Obstetrics, Hainan General Hospital, Hainan Affiliated Hospital of Hainan Medical University, Haikou, Hainan China; 4 Department of ICU, Hainan General Hospital, Hainan Affiliated Hospital of Hainan Medical University, Haikou, Hainan China

**Keywords:** Vagus nerve stimulation, Preeclampsia, Inflammation, α7nAChR, Cholinergic anti-inflammatory pathway

## Abstract

**Background:**

Preeclampsia is characterized by an excessive inflammatory response. Recent studies have shown that vagus nerve stimulation (VNS) has anti-inflammatory properties in vivo. This study aims to investigate whether VNS is safe for use during pregnancy and to explore the therapeutic potential and underlying mechanisms of VNS in PE.

**Methods:**

Pregnant Sprague-Dawley rats were randomly chosen to receive N-nitro-L-arginine methyl ester (L-NAME)-containing water (preeclampsia-like mouse model) or saline (normal pregnancy control) daily at gestational days 14.5–20.5. VNS and the α7nAChR antagonist methyllycaconitine citrate (MLA, 1 mg/kg/d) were given daily at the same time.

**Results:**

VNS decreased the high systolic blood pressure and urinary protein observed in the PE rats. In addition, VNS mitigated abnormal pregnancy outcomes. Moreover, VNS alleviated the inflammatory response by decreasing the levels of inflammatory cytokines. VNS significantly increased the expression of α7nAChR and attenuated the activation of NF-κB p65 in the placenta.

**Discussion:**

Our findings indicate that maternal VNS treatment is safe during pregnancy and has a protective effect in a pregnant rat model of preeclampsia induced by L-NAME.

## Background

Preeclampsia is a multifactorial and multisystemic disorder that occurs during pregnancy and that can lead to increased morbidity or mortality in both the mother and her unborn child [1]. A recent report shows that 2–8% of all pregnant women worldwide suffer from preeclampsia [2]. Preeclampsia is diagnosed by new onset hypertension (systolic pressure ≥ 140 mmHg and/or diastolic pressure ≥ 90 mmHg), which occurs most often after 20 weeks of gestation, and often accompanied by proteinuria [3]. The considerable variation in the onset, clinical manifestation, and severity of this pregnancy-specific disease creates major challenges in the balance of benefits and risks of delivery. Currently, the delivery of the placenta remains the only definitive cure for preeclampsia [1, 4].

Despite the severity of preeclampsia, the precise pathogenesis is not yet fully understood and is currently an area of active research [4]. A series of potential etiologies of preeclampsia, including endothelial dysfunction [5], excessive inflammation [6], immunological dysregulation [7] and oxidative stress [8], has been identified. Among these potential etiologies, the imbalance in pro- and anti-inflammatory networks has emerged as the one phenomenon that is most strongly related to the clinical symptoms and disease severity of preeclampsia. There is growing evidence that preeclampsia is closely linked to an abnormal inflammation response that reveals both locally in the placenta and systemically in the mother [9, 10]. Quenching or inhibiting inflammatory regulation pathways may be a real mechanism of the pathogenesis of preeclampsia. As such, therapies that selectively suppress the excessive inflammatory response without leading to broad immunosuppression are desired.

Preventing pro-inflammatory cytokine production through endogenous “neuro-immune” interactions has emerged as one such treatment. Tracey has revealed that the vagally mediated cholinergic anti-inflammatory pathway (CAP) is capable of acutely attenuating inflammation [11]. Electrical stimulation of the vagus nerve is known to have to have anti-inflammatory effects on multiple diseases, including ischemia reperfusion injury [12], sepsis [13] and rheumatoid arthritis [14]. Vagus nerve stimulation (VNS) is an effective nonpharmacologic approach for the treatment of inflammatory disease, but the use of VNS to inflammatory response following preeclampsia has not been thoroughly examined. Therefore, this research aimed to investigate whether VNS attenuate preeclampsia and to explore the underlying mechanisms involved using a preeclamptic model in rats.

### Sprague Dawley rats

An approximately of 6–8 weeks old (weight range: 250–300 g) Sprague-Dawley rats were obtained from the Medical Experimental Animal Center of Hainan. The experimental rats selected were fed with regular chow and water ad libitum under controlled conditions of temperature (22–24 °C), humidity (50–70%), and lighting (12:12-h light–dark cycle) [15]. The protocols for animal use and the procedures for the experiments described here were approved by the Animal Ethics Committee of Hainan Medical University, China (ratification NO 2020–185), and the experiments were performed according to the Guidelines for the Care and Use of Laboratory Animals.

### Experimental design

The female-male rats were placed in a ratio of 2:1, 7 days after acclimatization. The sperms presence in vaginal smears were used as a definition of gestational day (GD) 0. A single oral daily dose of 50 mg/kg L-NAME or distilled water from GD 14.5 to GD 20.5 was given to the experimental rats during pregnancy [16]. The rats were then put randomly into 8 groups, consisting 7–8 rats in a group as follows: Group 1, the normal control group, received distilled water. Group 2, the P + Sham control group, was treated as group 1 but subjected to sham stimulation daily for 7 days. Group 3, the P + VNS control group, was treated as group 1 but received vagus nerve stimulation for 7 days. Group 4, the P + VNS + MLA control group, was also treated as group 3 but MLA i.v. injection dose of 1 mg/kg/day was given daily for 7 days. Group 5, the PE group, was given L-NAME daily for 7 days. The PE + Sham group, Group 6, was treated as group 5 but exposed to sham stimulation. Group 7, the PE + VNS group, was treated as group 5 but with an additional daily vagus nerve stimulation for 7 days. Group 8, the PE + VNS+ MLA group, had group 7 treatment with an additional i.v. injection of MLA at a daily dose of 1 mg/kg/day for 7 days.

### VNS and sham surgery

Surgical protocols were previously described in detail [17]. In brief, the rats were anesthetized with isoflurane (5% in 100%O_2_) inhalation and maintained with a gas mask (2% isoflurane). The skin and muscles of the left cervical region were separated carefully, and then, the cuff was placed and fixed with a suture around the nerve. Continuous stimulation was delivered by a stimulator (BL-420, TME Technology Co., Ltd., Chengdu, China) on the cervical vagal trunk connected to a control module. The stimulation duration of the implanted VNS device was set to 30 min at the given frequency and was followed by an off-time of 5.5 h; this procedure was repeated 4 times per day from GD 14.5 to GD 20.5. The stimulation parameters are identical to those used in our previous study [18], namely, a stimulation frequency of 5 Hz, a low voltage of 3 V, a current of 1 mA and a pulse duration of 500 μs. For the sham rats, the surgery was performed in the same way, but the cuff electrode was not implanted.

### Measurement of blood pressure and heart rate

The tail-cuff technique with a BP-2000 Blood Pressure Analysis System (Visitech Systems Inc., North Carolina, USA) was used to test systolic blood pressure and heart rate in all the groups of rats on the GDs 12, 14, 16, 18 and 20. AS previously described, the cuff was placed around the tail, inflated to block blood flow, and then slowly deflated conferring to the internal programmer [19]. Each rat received 37 °C 30 min pre-warmed before each measurement was taken with each measured 3 times to obtain the average values.

### Urine protein concentration

On the GDs 13, 15, 17 and 19, the rats were housed separately for 24 h under the cages to collect urine samples. Urine samples were centrifuged at 2000 rpm for 15 min at 22 °C, and the supernatant was used for the analysis of protein levels by using a BCA protein assay kit (Thermo Fisher Scientific Inc., CN, Shanghai).

### Sample preparation

The experimental rats at GD 21, underwent cesarean section after anesthetize with isoflurane inhalation. The placentas and pups were dissected, inspected, counted, and weighed. The blood from the inferior cava vena were collected into plain bottles. Each placenta was placed in 4% paraformaldehyde fixation immediately after being washed by 0.9% NaCl, and embedded in paraffin, or placed in Trizol for real-time PCR, or frozen in liquid nitrogen for biochemical analysis and Western blot analysis. All the experimental maternal and neonatal rats were euthanized with a lethal dose of isoflurane (10–20%) inhalation immediately after the procedure.

### Histology and immunohistochemistry

The placental specimens were placed in 4–5 μm thick paraffin sections, stained with hematoxylin and eosin by the H&E protocol standardization. An optical microscope (Olympus BX51, Tokyo, Japan) was used to observe and photographed. These sections were detected by an eligible and blinded pathologist to assess the extent of pathological changes.

The α7 nicotinic acetylcholine receptor (α7nAChR) and nuclear factor-κB (NF-κB) p65 in the placentas were assessed by Immunohistochemical analyses performed. An alcohol gradient was used to deparaffinized and hydrated the placental sections selected for immunostaining. The sections were then incubated in a 1:1000 dilution of primary antibodies against α7nAChR (cat. no. ab10096; Abcam, MA, USA) and NF-κB p65 (cat. no. ab207297; Abcam, MA, USA) overnight at 4 °C. Goat anti-mouse IgG secondary antibodies were added to the sections at 37 °C and incubated for 30 min on the following day. A 3,3′-diaminobenzidine solution (Leica Microsystems, Shanghai, China) was used to dye the sections, counterstained with hematoxylin, dehydrated with ethanol and mounted in synthetic resin. The Image-Pro Plus v6.0 software (Media Cybernetics, Inc., Bethesda, Maryland) was used for the expression of α7nAChR and NF-κB p65 examination by comparing positively labeled areas and total areas. The primary antibody was replaced with goat serum for the negative controls.

### Enzyme-linked immunosorbent assay (ELISA) method

AS previously described, the concentrations of TNF-α, IL-1β, IL-6, IL-10 and IFN-γ in the maternal serum and placental homogenate were determined according to the manufacturer’s instructions by ELISA kits (R&D Systems, Minneapolis, MN) [20]. The analysis was performed in duplicate using a Bio-Plex™ system (Luminex Bio-Plex™ 200 System, Bio-Rad).

### Measurement of gene expression by quantitative polymerase chain reaction (qPCR)

Total RNA was extracted from the placental tissues by using TRIzol (Invitrogen, Carlsbad, CA, USA). The RNA concentration was determined by NanoDrop 2000 analysis (NanoDrop Products, DE, USA). The SuperScript™ VILO™ cDNA Synthesis Kit (Invitrogen, USA) was used for reverse transcription, and the TaqMan Gene Expression Master Mix (Applied Biosystems) was used for qPCR according to the manufacturer’s protocol. The qPCR parameters and all the primers for the amplification of the studied genes (α7nAChR, NF-κB p65 and β-Actin) were used according to Kong et al. [21] Thermal cycler settings were 95 °C for 5 min, then 40 cycles of 95 °C for 20 s and 60 °C for 40 s, and 72 °C for 8 min. The primer sequences (5′ to 3′) for α7nAChR, NF-κB p65 and β-Actin were as follows (forward and reverse, respectively): α7nAChR (ACCTCGTGTGATCCAAAGCC and GGTTTCCTCTTGCTCAGGGT); NF-κB p65 (CGACGTATTGCTGTGCCTTC and TGAGATCTGCCCAGGTGGTAA) and β-Actin (CCTCTATGCCAACACAGTGC and GTACTCCTGCTTGCTGATCC). β-Actin was used as the housekeeping gene. Data analysis was carried out using the 2^-ΔΔCT^ method.

### Statistical analysis

The experimental data are reported as the mean ± standard error of the mean. One-way analysis of variance (ANOVA) was done for the comparisons between multiple groups, the least significant difference (LSD) post hoc test or Dunnett’s test were followed. SPSS software, version 16.0 (SPSS Inc., Chicago, IL, USA) were used for the data analyzes. *P*-values of 0.05 were considered to indicate a statistically significant difference.

## Results

### Effect of VNS on the blood pressure and heart rate of rats with L-NAME-induced preeclampsia

**Fig. 1:**
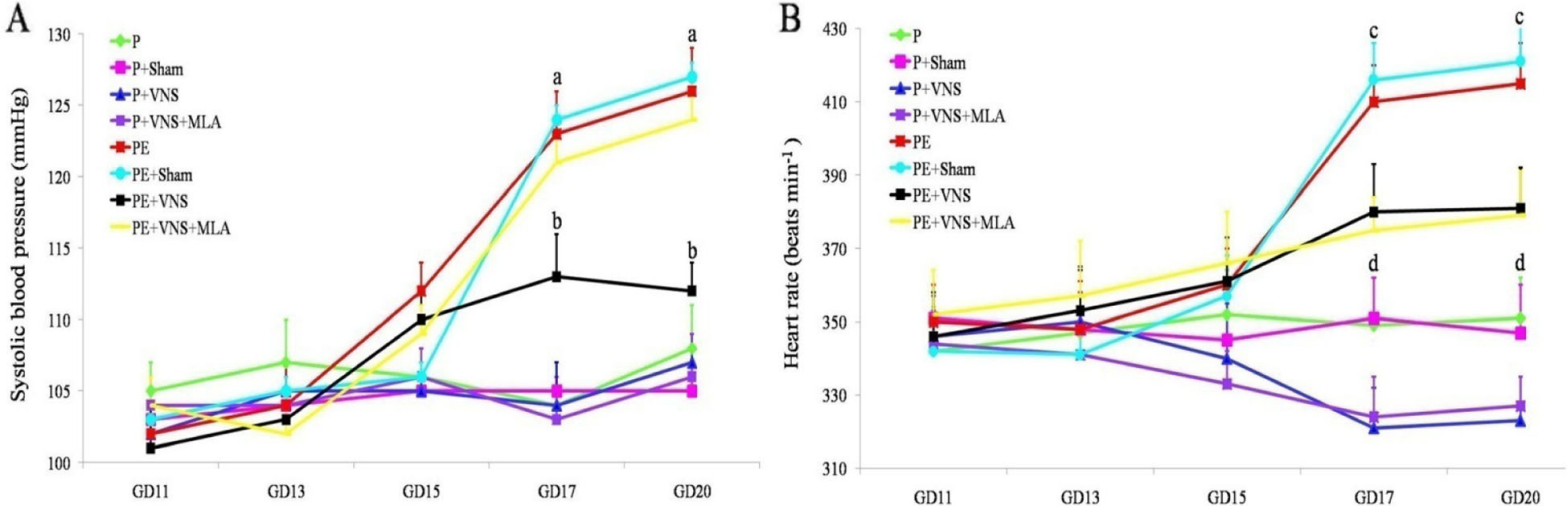
Effect of VNS on the blood pressure and heart rate of rats with L-NAME-induced PE. Systolic blood pressure (**a**) and heart rate (**b**) of pregnant rats treated or not (control) with L-NAME or L-NAME plus VNS in different periods of pregnancy. Data are presented as Mean + SEM. **a** *p* < 0.05, the PE, PE+ sham and PE+ VNS and PE + VNS + MLA groups compared to the P group on GD 17 and GD 20; **b** *p* < 0.05, the VNS group compared to the P, PE, PE+ sham and PE + VNS + MLA on GD 17 and GD 20; **c** *p* < 0.05, the PE and PE+ sham groups compared to and PE + TaVNS and PE + VNS + MLA groups on GD 17 and GD 20; **d** *p* < 0.05, the P and P+ sham groups compared to and P+ VNS and P + VNS + MLA groups on GD 17 and GD 20

The baseline SBP values were not significantly different among the groups on GD 11 and GD 13 (*P* > 0.05). On GD 15, the SBP was increased in the L-NAME-treated group, but did not show significant changes compared with the water-treated group (*P* > 0.05). On GD 17 and GD 20, we observed a significantly lower SBP in the L-NAME-treated rats that were treated with VNS than that in the untreated rats. Moreover, the SBP was not significantly different between the water-treated groups (*P* > 0.05, data not shown) at that time (Fig. 1a).

In accordance with the SBP, the average baseline HR was similar among the groups (Fig. 1b). L-NAME administration induced a rapid and progressive increase in the HR beginning on GD 17. The rats that received L-NAME and underwent VNS showed an increased HR compared to the control rats but a reduced HR compared to the rats that received L-NAME alone. We also observed that concurrent treatment with VNS and MLA significantly attenuated the elevated HR in the L-NAME-treated rats. Interestingly, among the water-treated groups, the P + VNS or P + VNS + MLA groups exhibited a significantly lower HR than the P and P + sham groups on GD 18 and GD 20 (*P* < 0.01).

### Effect of VNS on urinary protein in rats with L-NAME-induced preeclampsia

**Fig. 2:**
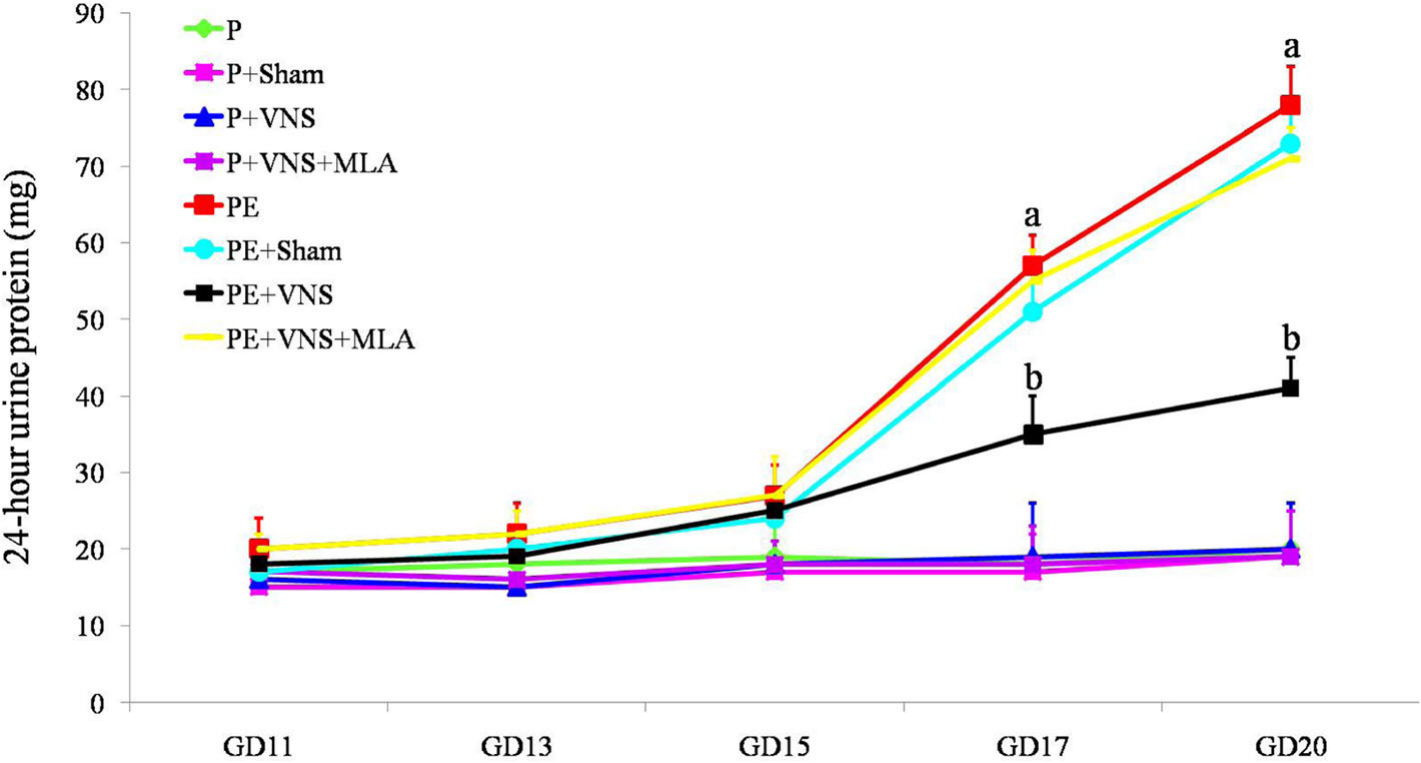
Effect of VNS on urinary protein in rats with L-NAME-induced PE. VNS ameliorated urinary protein in the L-NAME induced PE like rats. The 24-h urinary protein was measured on GDs 11, 13, 15, 17, and 20. Data are presented as Mean + SEM. **a** *p* < 0.05, the PE, PE+ sham and PE+ VNS and PE + VNS + MLA groups compared to the P group on GD 17 and GD 20; **b** *p* < 0.05, the VNS group compared to the P, PE, PE+ sham and PE + VNS + MLA on GD 17 and GD 20

At the beginning of the experiment, the degree of proteinuria was not significantly different (*P* > 0.05) between the groups. The increase in proteinuria after L-NAME treatment was significantly enhanced on GD 17 and 20. VNS administration show a reduction in proteinuria of the L-NAME-treated rats. Moreover, the 24-h urinary protein level was significantly higher in the PE + VNS + MLA rats than in the PE + VNS rats (*P* < 0.05). The 24-h urinary protein level did not significantly vary throughout pregnancy in the water-treated groups (*P* > 0.05) (Fig. 2). The data for the P + sham, P + VNS and P + VNS + MLA groups are not shown.

### Effects of VNS on the morphological changes in the placenta in rats with L-NAME-induced preeclampsia

**Fig. 3:**
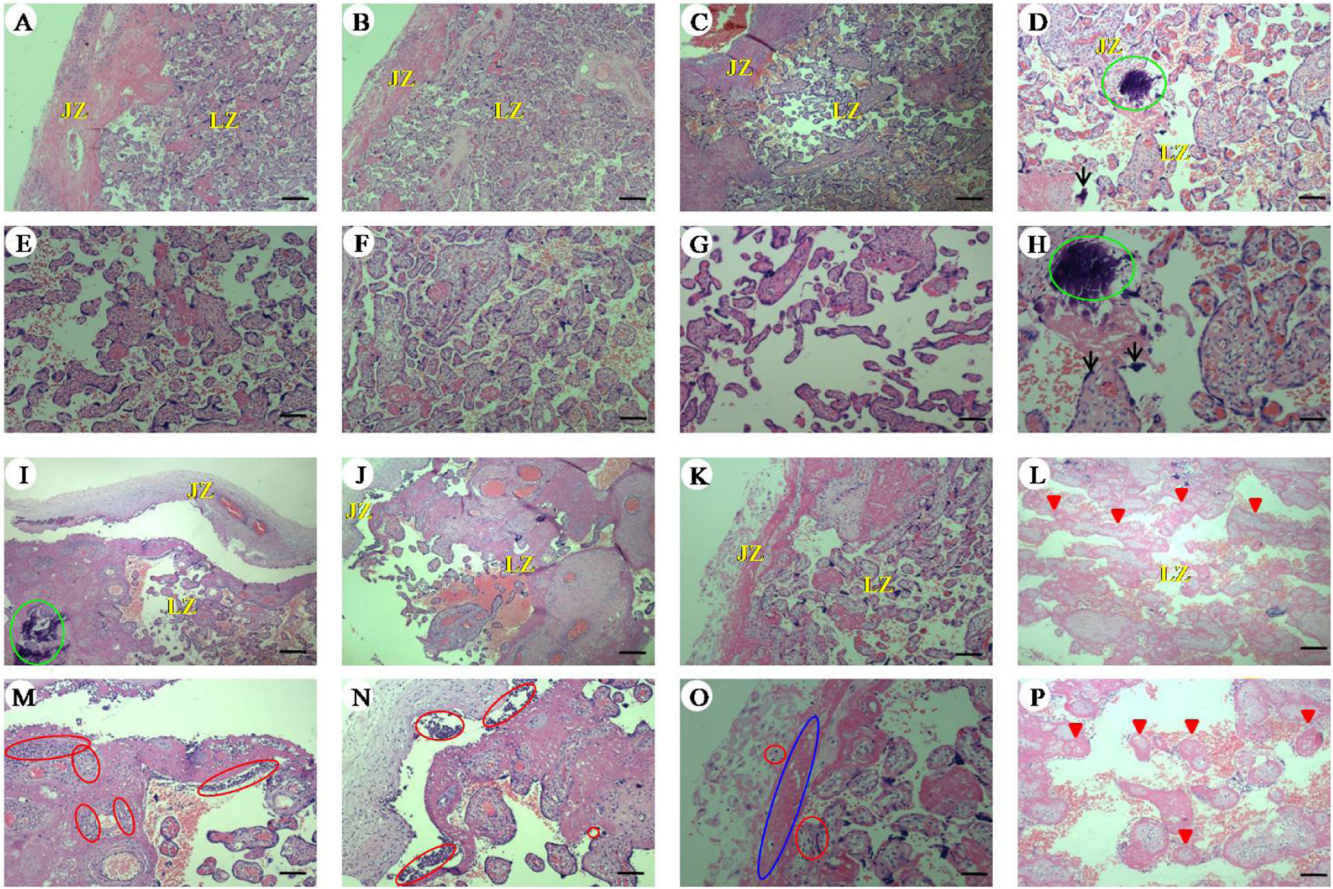
Effects of VNS on the morphological changes in the placenta in rats with L-NAME-induced PE. Changes of placenta structure in pregnant rats (GD 21). **a**-**h** Histopathological images of placenta in pregnant rats of (**a** and **e**) P, (**b** and **f**) P + Sham, (**c** and **g**) P + VNS, (**d** and **h**) P + VNS+ MLA, (**i** and **m**) PE, (**j** and **n**) PE + Sham, (**k** and o) PE + VNS and (**l** and **p**) PE + VNS+ MLA groups. Histological section at low (**a**-**d** and **i**-**l**) and high (**e**-**h** and **m**-**p**) magnification. LZ: Labyrinth; JZ: Junctional zone. Arrows: syncytiotrophoblast nodules; Red triangle: villous infarction. Red circle: inflammatory cell infiltration; Green circle: calcification; Blue circle: fibrin-like substance deposition. (H&E; low magnification, × 40; scale bar = 30 μm; high magnification, × 100; scale bar = 75 μm)

As shown in Fig. 3, optical microscopic observation (H&E staining) revealed slight calcification and a few syncytiotrophoblast nodules of labyrinth of the placenta in the water-treated groups. The labyrinth and junctional zone of the placenta were seriously impaired in the PE and PE + Sham groups, as indicated by substantial inflammatory cell infiltration, large-scale villous infarction and fibrin-like substance deposition. Chronic VNS reversed the detrimental changes in the placenta in the PE + VNS rats. However, the morphological characteristics of placentas in the PE + VNS + MLA group did not differ from those in the PE and PE + Sham groups.

### Effects of VNS on pregnancy outcomes in rats with L-NAME-induced preeclampsia

We chose various indexes to assess pregnancy outcomes, mainly maternal and placental weight, pup weight and live pup number (Table 1). There were no differences in maternal and placental weight among the various groups. L-NAME administration significantly lowered the weight of the pups and the number of living pups compared to those of the pregnant control groups. In addition, VNS treatment during pregnancy significantly alleviated the L-NAME-induced an increase n in the number of resorbed fetuses, but the effects of VNS were abolished by MLA.

**Table 1:** The effects of VNS treatment on pregnancy outcomes in L-NAME induced PE rats on GD 21

Group (*n* = 8)	Live fetuses	Resorbed fetuses	Maternal weight (g)	Fetal weight (g)	Placental weight (g)
P	15.22 ± 0.35	0.82 ± 0.35	401.21 ± 11.37	4.77 ± 0.12	0.56 ± 0.021
P + VNS	14.19 ± 0.54	0.91 ± 0.61	398.20 ± 15.64	4.69 ± 0.06	0.52 ± 0.023
P + Sham	15.70 ± 0.61	0.89 ± 0.38	400.41 ± 13.12	4.70 ± 0.09	0.54 ± 0.035
P + VNS + MLA	15.42 ± 0.39	0.95 ± 0.21	397.03 ± 14.31	4.72 ± 0.11	0.51 ± 0.041
PE	10.18 ± 0.84^*/#^	2.01 ± 0.11^*/#^	396.21 ± 13.72	3.58 ± 0.08^*/#^	0.50 ± 0.027
PE + VNS	13.16 ± 0.68^*^	1.53 ± 0.51^*^	399.21. ± 12.03	4.41 ± 0.13^*^	0.53 ± 0.033
PE + Sham	10.41 ± 0.75^*/#^	2.15 ± 0.68^*/#^	400.48 ± 14.90	3.67 ± 0.10^*/#^	0.52 ± 0.042
PE + VNS + MLA	11.17 ± 0.42^*/#^	1.89 ± 0.49^*/#^	398.92. ± 13.82	3.89 ± 0.07^*/#^	0.55 ± 0.019

Data are analyzed by One-way ANOVA, and presented as Mean + SEM. *P < 0.05 vs P, P + VNS, P + Sham and P + VNS + MLA; ^#^*P* < 0.05 vs PE + VNS group

### Effects of VNS on the levels of proinflammatory cytokines in both the serum and placenta in rats with L-NAME-induced preeclampsia

**Fig. 4:**
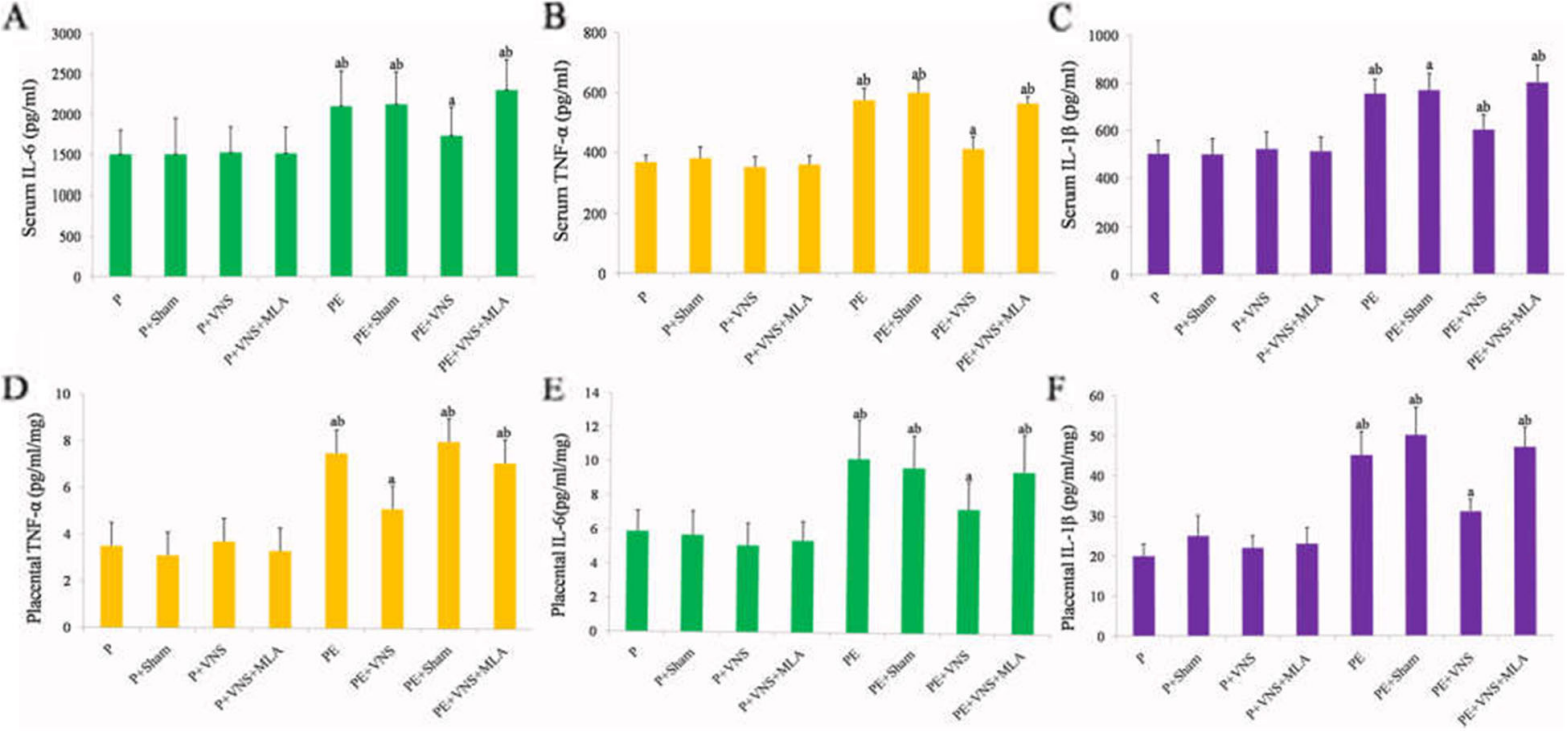
Effects of VNS on the levels of proinflammatory cytokines in both the serum and placenta in rats with L-NAME-induced PE. VNS treatment attenuated inflammatory cytokines in both plasma and placenta in L-NAME induced PE rats. Serum TNF-α (**a**), IL-1β **(b**) and IL-6 (**c**), placental TNF-α (**d**), IL-1β (**e**) and IL-6 (**f**) were measured in different group on GD 20. Data were presented as Mean + SEM. ^a^*P* < 0.05 vs P, P + VNS, P + Sham and P + VNS + MLA; ^b^*P* < 0.05 vs PE + VNS group

We measured the serum and placental protein concentrations of TNF-α, IL-1β, IL-6, IL-10 and IFN-γ in the 8 groups. In the PE and PE + Sham rats, TNF-α was significantly upregulated in both the serum and placenta compared with the levels in the water-treated rats, and the upregulation was significantly inhibited by VNS; this trend was also observed for IL-1β and IL-6. However, the inhibitory effects of VNS were significantly abrogated by MLA (Fig. 4). The levels of IL-10 and IFN-γ in the serum and placenta did not vary significantly across different groups (*P* > 0.05, data not shown).

### VNS increased placental α7nAChR mRNA and protein expression in rats with L-NAME-induced preeclampsia

**Fig. 5:**
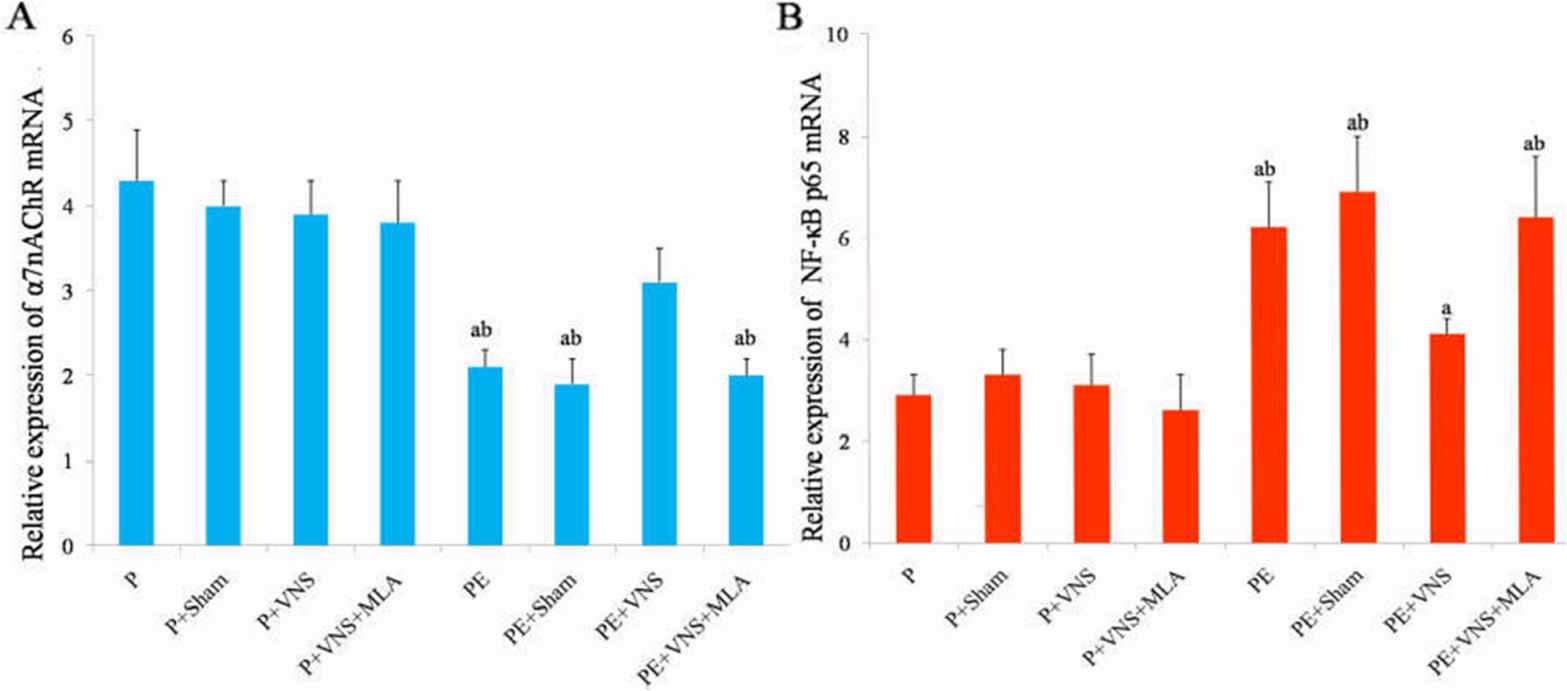
The expression of α7nAChR (**a**) and NF-κB *65* (**b**) mRNA in the placenta as determined using qPCR. Data were presented as Mean + SEM. ^a^*P* < 0.05 vs P, P + Sham, P + VNS and P + V NS + MLA; ^b^*P* < 0.05 vs PE + VNS group

The real-time PCR results showed that the α7nAChR mRNA expression was no statistical difference between the P + VNS group and the other water-treated groups. The downregulated α7nAChR mRNA levels following L-NAME treatment were elevated by VNS treatment but not by concurrent treatment with VNS and MLA. No statistically significant differences were observed among the P, P + sham, P + VNS + MLA, PE, PE + sham and PE + VNS + MLA groups (*p* > 0.05) (Fig. 5a).

**Fig. 6:**
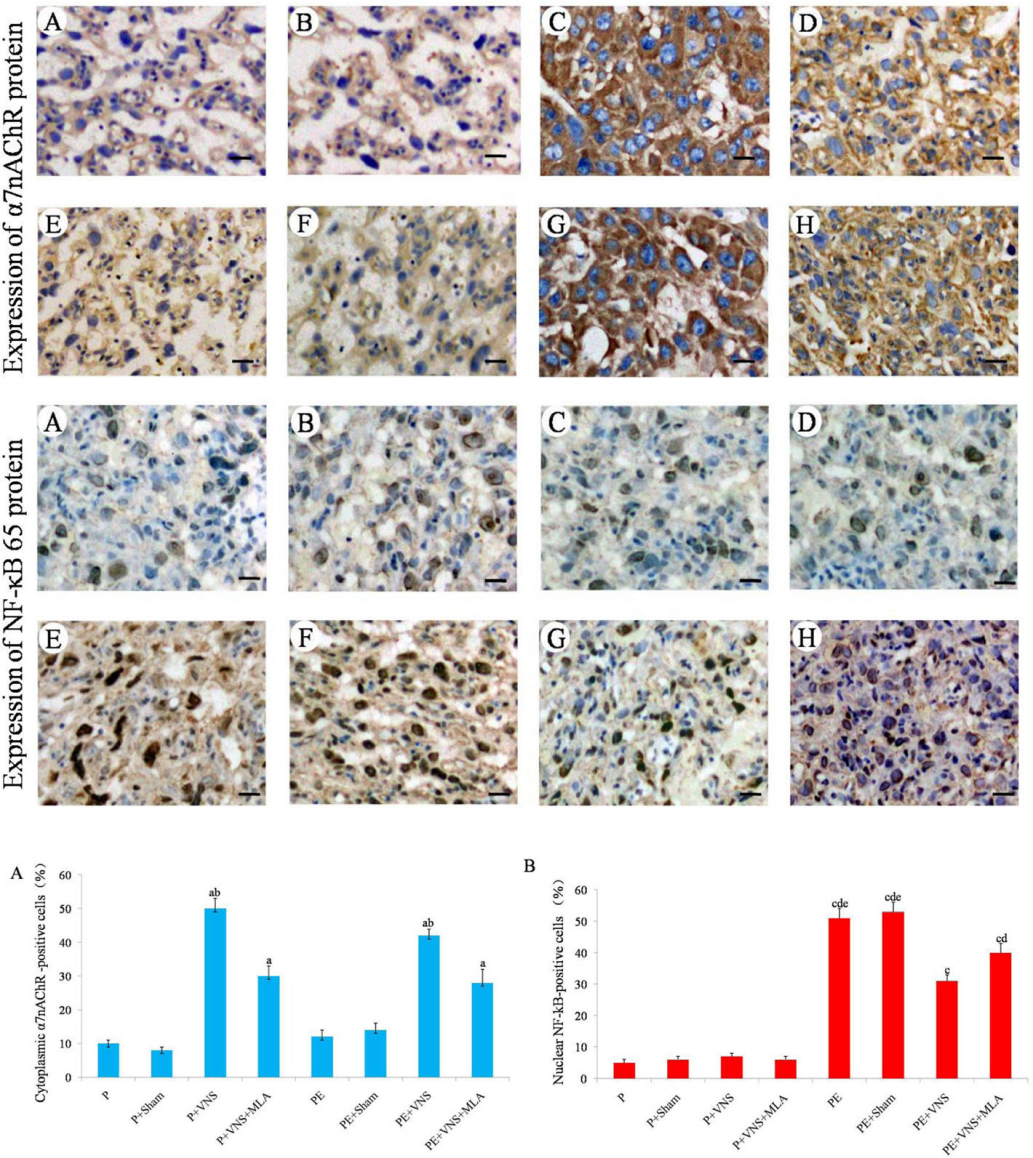
The expression of α7nAChR and NF-κB 65 protein in the placenta as determined using immunohistochemistry. **a**: P group; **b**: P + Sham group; **c**: P + VNS group; **d**: P + VNS + MLA group; **e**: PE group; **f**: PE + Sham group; **g**: PE + VNS group; **h**: PE + VNS + MLA group. Data were presented as Mean + SEM. ^a^*P* < 0.05 vs P, P + Sham, PE and PE + Sham groups; ^b^*P* < 0.05 vs P + VNS + MLA and PE + VNS + MLA groups. ^c^*P* < 0.05 vs P, P + Sham, P + VNS and P + V NS + MLA groups; ^d^*P* < 0.05 vs PE + VNS group ^e^*P* < 0.05 vs PE + VNS + MLA groups. Original magnification: × 400; scale bar =30 μm

The immunohistochemical assay results also revealed that α7nAChR was mainly localized in the cytoplasm of placental labyrinth zone from the VNS-treated groups. The α7nAChR protein expression was significantly higher in the P + VNS group than in the P and P + sham groups (*p* < 0.05), and its levels were even higher in the PE + VNS group than that in the PE and PE + sham groups. Moreover, the effects of VNS were significantly blocked by MLA (*p* < 0.05) (Fig. 6).

### VNS increased placental NF-κB 65 mRNA and protein expression in rats with L-NAME-induced preeclampsia

The placental NF-κB 65 mRNA and protein expression levels showed no significant difference (*P* > 0.05) among the water-treated groups. Maternal exposure to L-NAME significantly increased (*P* < 0.05) the level of NF-κB 65 mRNA in the placenta. L-NAME-induced placental NF-κB 65 mRNA expression was attenuated (*P* < 0.05) by VNS treatment during gestation (Fig. 5b).

Immunohistochemical assay results also indicated that the level of placental nuclear NF-κB p65 was significantly increased (*P* < 0.05) in rats treated with L-NAME (Fig. 6), and this level was decreased (*P* < 0.05) by VNS administration. However, MLA supplementation dramatically inhibited the VNS-induced decreases in NF-κB p65 mRNA and protein expression (*P* < 0.05).

## Discussion

In this study, we found that VNS treatment improved adverse pregnancy outcomes, such as decreasing the high SBP and HR, reducing the urinary protein excretion, promoting recovery from placental injury, and suppressing inflammation in rats with L-NAME-induced preeclampsia. Moreover, VNS also increased placental α7nAChR expression and effectively inhibited placental NF-κB p65 activation. Meanwhile, the protective effects of VNS could be blocked by systemic pretreatment with a specific α7nAChR antagonist.

Generalized activation of the inflammatory pathways is thought to play a role in the pathogenesis of preeclampsia [22]. The increased levels of inflammatory cytokines in the amniotic fluid and/or cord blood during preeclampsia are involved in adverse maternal and neonatal outcomes [23]. Taken together, these studies suggest that inflammatory pathways are not only associated with preeclampsia but also may be responsible for adverse neonatal outcomes. There is growing evidence indicating that administering anti-inflammatory agents to the mother may ameliorate the adverse perinatal outcomes of preeclampsia. The treatment of rats in preeclampsia with anti TNF-α antibodies attenuated hypertension and decreased IL-6 and sVCAM-1 levels [24]. As an anti-inflammatory constituent of the herb, uncaria rhynchophylla can suppress inflammation and mitigating preeclampsia-like symptoms in a rat model [19]. There is some evidence suggesting that in addition to anticoagulation, the potential effects of low molecular weight heparin in preventing preeclampsia progress are mediated by suppressing inflammation [25].

The cholinergic anti-inflammatory pathway (CAP) bridges the immune and nervous systems [11] and plays multi-effect roles in modulating inflammation. These pathways affect the afferent sensory nerves of the solitary nucleus, which in turn activates the efferent vagus nerve and promotes the release of acetylcholine (Ach). Then, ACh stimulates its receptor, α7nAChR, which results in the inhibition of a key molecular mediator of inflammation [11]. The CAP can also be activated by VNS or α7nAChR stimulation. Previous research has shown that treatment with α7nAChR agonists, such as nicotine [15] and choline [26], relieved preeclampsia symptoms and improved adverse fetal outcomes, including fetal loss and intrauterine growth restriction, in pregnant rats administered LPS. Therefore, we aimed to explore whether the stimulation of efferent vagus nerve directly regulate the inflammatory response to L-NAME during pregnancies in rat model.

L-NAME is a nonspecific nitric oxide synthase inhibitor that has been confirmed to increase the vascular response, block the relaxation of the vascular endothelium and form a narrow spiral artery. Many studies have shown that injection of pregnant rats with L-NAME exhibit preeclampsia-like symptoms [27]. In the present study, a rat model of preeclampsia was successfully established and suitable for further analysis. The results indicated that L-NAME led to an increase in the systolic blood pressure and heart rate during pregnancy. Furthermore, substantial proteinuria, decreased fetal weight, increased embryonic resorption, and high proinflammatory cytokine production in the maternal and placenta of rats were induced.

VNS was a common treatment for epilepsy in more than 100,000 patients, and it is generally well tolerated [28]. Recently, VNS has been found to provide protection against systemic inflammation injury in rats [12]. VNS regulates the CAP and inhibits cytokine synthesis, and subsequent prevention of organs or tissues injury. In addition, VNS is associated with decreased heart rate variability, which is a diagnostic tool in the detection of autonomic impairment in a rat model of inflammatory disease [29]. This study shows that chronic VNS also causes an attenuation of SBP elevation during the development of hypertension in preeclampsia. The effectiveness of VNS’s blood pressure-lowering effects depends on several factors, including the stimulus parameters, position, administration route, and experimental model [30].

In addition, our data further demonstrated that MLA failed to block the effect of VNS on the lower heart rate in VNS-treated animals. This observation suggests that the cardioprotective effect of VNS is independent of its effect on heart rate reduction, which is consistent with a previous study [31]. The efficacy of heart rate reduction by VNS is considered to be contributed to the modulation of the autonomic nervous system, which is related to increased sympathetic activity [32]. Another possibility is that the cardiac electrophysiological regulation by VNS may be modulated through muscarinic acetylcholine receptors (mAChR), since the activation of muscarinic receptors in VNS-mediated cardioprotection was found in the previous study [33].

Proteinuria is not essential to diagnosis but is related to disease severity and fetal outcomes in preeclampsia. In the study, we found proteinuria and fetal growth restriction in L-NAME-treated rats, which is consistent with previous studies [34]. However, mild proteinuria was observed in the VNS-treated rats. We also identified lower fetus weights in the L-NAME-treated groups, which concur with published research papers [35]. Partial reversal of growth retardation was observed in the VNS group, where the weight of the fetuses was similar to that of fetuses from the control group.

The placenta of the rats that received only L-NAME showed histopathological changes including inflammatory cell infiltration, villous infarction and fibrin-like substance deposition. But these changes were maintained at mild intensity after VNS. These data indicate that VNS improved the placental pathology, suggesting that VNS may provide protection for the fetus during preeclampsia.

Previous research has shown that CAP is involved in the limitation of inflammatory responses, which depends on α7nAChR in animal models. α7nAChR was initially found in the nervous system; however, its expression in nonneuronal cells, such as macrophages, endothelial cells, smooth muscle cells and the placenta [36], was only recently discovered. α7nAChR expressed in the placenta may have an anti-inflammatory role and inhibit the activation of endothelial cells, which are involved in the pathophysiology of preeclampsia [27]. In this study, we found that VNS selectively suppressed the pro-inflammatory cytokines production in the serum and placenta but left anti-inflammatory cytokines undisturbed. Additionally, the lower mRNA and protein levels of α7nAChR were observed in the placentas of L-NAME groups. Moreover, we also found that VNS significantly increased the level of α7nAChR expression. These data show that it is reasonable to propose that the inhibition of placental and peripheral cytokine production may be the result of VNS inducing the activation of α7nAChR.

It is well known that NF-κB plays a central role in the expression of genes involved in immune and inflammatory responses [37]. Human study have demonstrated increased NF-κB activation and increased NF-κB activation in vessels and placenta in women with preeclampsia [38]. NF-κB inhibition leads to the suppression of TNF-α, TGF-β1, and IL-10 production [39]. Thus, the pathways involved in NF-kB activation are likely targets for reducing inflammation. The anti-inflammatory properties of α7nAChR-mediated CAP have been attributed to the inhibition of NF-κB activation. NF-κB activation was represented by the ratio of the cytoplasmic to nuclear localization of the NF-κB p65 subunit. Research has shown that VNS exerts anti-inflammatory effects on monocytes challenged with the release of the proinflammatory cytokine TNF-α by partially inhibiting NF-κB p65 activation. VNS treatment attenuated the L-NAME-induced increase in placental nuclear NF-κB p65, while MLA significantly antagonized these effects.

## Conclusions

Our research showed that VNS improved PE symptoms and inhibited pro-inflammatory cytokines in a rat model of PE. These findings revealed a novel therapeutic use of VNS for preventing excessive pro-inflammatory cytokines during pregnancy complications. VNS is a non-pharmacological treatment. VNS has emerged as a potential non-pharmaceutical treatment strategy with anti-inflammatory and anti-hypertensive properties. However, different stimulation parameters and sites have been shown to exert distinct therapeutic effects. In this study, we only verified the current stimulation parameters of left-side VNS and found that the heart rate is regulated in normal pregnant rats. Further studies will aim to explore the best stimulation parameters and the underlying mechanisms. Furthermore, the beneficial effects of HRV biofeedback on preeclampsia symptoms and cardiovascular system by increasing vagal tone need to be elucidated in further research.

## Data Availability

The datasets used and/or analysed during the current study are available upon reasonable request from the corresponding author.

## Abbreviations

VNS: Vagus nerve stimulation
L-NAME: NG-nitro-L arginine methyl ester
GD: Gestational day
NF-kB: Nuclear factor-kB;
ELISA: Enzyme-linked immunosorbent assay
α7nAChR: Nicotinic acetylcholine receptor type 7
MLA: Methyllycaconitine citrate
CAP: Cholinergic anti-inflammatory pathway
Ach: Acetylcholine
